# Healthcare Access Dynamics and Characteristics of Foreign Nationals Using Emergency Departments: A Retrospective Study from Türkiye

**DOI:** 10.3390/healthcare14040488

**Published:** 2026-02-14

**Authors:** Gokhan Taskin, Murat Aysin

**Affiliations:** 1 Department of Emergency, Faculty of Medicine, Balikesir University, Balikesir 10020, Türkiye; 2 Department of Public Health, Faculty of Medicine, Balikesir University, Balikesir 10020, Türkiye; murataysin@gmail.com

**Keywords:** immigrant health, emergency department, healthcare services, foreign national patients

## Abstract

Background/Objectives: Türkiye is located at the intersection of major international migration routes and plays a critical role in global migrant health. The growing immigrant and refugee population has introduced new challenges to healthcare systems (HCSs), particularly in emergency departments (EDs). This study aims to address a gap in the literature by evaluating the reasons foreign nationals present to the ED, their diagnostic distribution, and access to healthcare services in relation to sociodemographic characteristics. Methods: This retrospective study included foreign nationals who presented to the Emergency Department of Balikesir University Hospital between January 2020 and June 2025. Sociodemographic and clinical data were obtained from the hospital information management system. Data analysis was performed using IBM SPSS Statistics 27.0. Descriptive statistics were used to summarize demographic and clinical characteristics, including gender, nationality, admission period, diagnosis groups, laboratory testing, consultations, and patient outcomes. Results: A total of 6366 foreign nationals were included. Of these, 66.4% were female and 33.6% were male, with a mean age of 31.9 years for females and 26.8 years for males. Syrians constituted the largest group (50%). ED visits occurred most frequently in spring (30.1%) and peaked in 2024. The majority of patients (99.3%) were managed as outpatients. The most common diagnoses were internal medicine-related conditions (36.1%) and genitourinary emergencies (32.2%). Consultations were most frequently requested from the obstetrics and gynecology department (21.2%). Overall, 92.9% of patients were discharged from the ED. Conclusions: The findings suggest that foreign nationals often use emergency services as an alternative to primary healthcare. Low rates of laboratory and imaging utilization indicate that most ED visits were for non-urgent and uncomplicated conditions. Factors such as language barriers, communication difficulties, prolonged observation times, and limited social support may contribute to this pattern. This study provides comprehensive local data on the emergency care utilization of foreign nationals in Türkiye and offers valuable insights for healthcare planning and policy development.

## 1. Introduction

Globally, migration movements are increasing due to social dynamics such as climate change, political instability, economic inequalities, and conflicts, creating serious pressures on health systems. According to International Organization for Migration data, 272 million people were international immigrants in 2019, and this number is expected to increase further in the coming years [1]. The healthcare needs of immigrants directly impact the healthcare infrastructure of host societies and increase the complexity of service delivery [2].

Due to its geographical location and long borders, Türkiye is both a transit country and a final destination for many migrants [3]. Studies on foreign nationals’ access to healthcare services in Türkiye reveal the existence of multidimensional barriers such as a lack of integration into the system, language barriers, a lack of social security, and poor health literacy. It has been found that the “fit” dimension of migrants’ access to the healthcare system (HCS) is related not only to inadequate infrastructure or healthcare policies, but also to migrants’ level of knowledge, social capital, and the flexibility of accepting institutions [3,4].

The use of Emergency Departments (EDs) by foreign nationals is an increasingly focused area of the literature. EDs are often the first point of contact with the HCS for many foreign nationals; in this context, emergency medicine specialists directly encounter the clinical presentations and HCS barriers of this population. Studies systematically examining differences in emergency department use between foreign nationals and the native population have reported that foreign nationals may use emergency departments for less urgent conditions [5]. Another study of a similar nature found that foreign national-related patients had lower emergency department admission rates, that referral rates differed, and that certain groups had frequent visits [6]. A study examining ED visits by undocumented immigrants found that 61% of these visits were for preventable or primary care-manageable conditions, suggesting that immigrants view EDs as a “lifeline” for unnecessary use [7,8].

The barriers foreign nationals face in accessing healthcare are multifaceted. A recent study that comprehensively examined the difficulties immigrants encounter in accessing emergency healthcare services showed that legal barriers, financial constraints, and language and cultural differences are significant obstacles to undocumented immigrants accessing emergency care services [9]. Additionally, it has been noted that there is a strong relationship between the barriers to access perceived by immigrants themselves (e.g., unfamiliarity with the HCS, fear, and communication problems) and their healthcare utilization behavior [10].

The international literature examining the ED utilization profiles of immigrants has shown that sociodemographic characteristics such as age, gender, and social status play an important role in patterns of emergency healthcare use.

In addition to socio-demographic factors, the clinical profile of ED visits among immigrants also varies across settings. Previous studies have reported that infections, trauma, and gastrointestinal disorders are among the most common reasons for emergency department visits in undocumented immigrant populations, and that a significant proportion of patients, as much as 81%, are discharged after evaluation [7].

Moreover, comparative studies have suggested differences between immigrants and native populations in the frequency and urgency of ED admissions. Some reports indicate that immigrants may be brought to the ED less frequently and have lower admission rates than non-immigrant patients, while certain subgroups demonstrate frequent repeat visits [6].

Balikesir province is in a privileged position compared to other cities in our country due to its geographical location and geophysical structure. A large part of Balikesir Province is located in the South Marmara Region, while another part extends into the Aegean Region. Balikesir Province’s role as a bridge between the Marmara and Aegean regions and the presence of the Greek island of Lesbos opposite the district of Ayvalık have made it an important migration route for irregular immigrants seeking to reach Europe illegally from Türkiye [11]. In this study, regular/irregular migrants and individuals who are not citizens of the Republic of Turkey and whose nationality is recorded as not being Turkish in the hospital information management system, such as refugees, asylum seekers, tourists, or undocumented migrants, are referred to as “foreign nationals”. In this study, regular/irregular migrants and individuals who are not citizens of the Republic of Turkey and whose nationality is recorded as not being Turkish in the hospital information management system, such as refugees, asylum seekers, tourists, or undocumented migrants, are referred to as “foreign nationals”. In this context, a retrospective review of the demographic characteristics, reasons for referral, and diagnosis and treatment processes of foreign national patients who applied to the Emergency Department of Balikesır University Faculty of Medicine between 2020 and 2025 will fill gaps in the literature and be valuable for emergency medicine practice and public health. Unlike many previous reports focusing on specific refugee groups or short time periods, this study provides a comprehensive six-year ED-based profile of foreign nationals, including diagnostic categories, consultation patterns, laboratory utilization, observation time, and clinical outcomes, thereby offering operational insights for emergency care planning. Through this study, we aim to create an important data foundation for health service planning, resource management, and policy recommendations regarding foreign nationals’ health by identifying access to emergency health services, service usage patterns, and systemic barriers.

## 2. Materials and Methods

### 2.1. Research Model

This study was designed as a descriptive retrospective cross-sectional study, in which the existing situation is identified and described in its current state. In this approach, the phenomenon under investigation is examined as it naturally occurs, without any manipulation or control over the variables [12].

### 2.2. Research Group

This research used a census (complete enumeration) approach, including all relevant individuals over a six-year period from January 2020 to July 2025. The complete enumeration method is an effective data collection method that eliminates sampling error by reaching the entire population [13]. Foreign nationals were defined as patients registered in the hospital information management system with non-Turkish nationality at the time of the ED visit. The dataset did not allow reliable differentiation between immigrants, refugees, asylum seekers, tourists, or undocumented individuals. Nationalities represented by fewer than 50 individuals (12 nations, including India, China, Greece, Bulgaria, etc.) were grouped under the ‘Other’ category, which comprised 433 participants in total, to ensure analytical clarity and maintain the statistical robustness of subgroup comparisons.

### 2.3. Data Collection

In the study, a data recording form created by the researchers was used to obtain demographic and clinical information about the participants. The form included variables such as gender, age, nationality, period of application, diagnosis group, laboratory tests, consultation status, observation period, and outcome. Diagnosis groups were created based on the primary ED diagnosis recorded in the hospital information management system. Diagnoses were initially coded using the routine ICD-10 diagnostic coding structure, and subsequently aggregated into broader clinical categories for descriptive reporting. The aggregation was performed by the authors prior to analysis, and each ED visit was assigned to a single category based on the primary diagnosis. The data were retrospectively retrieved from the hospital information management system and analyzed based on the existing records without any intervention. Mortality data (in-hospital or post-discharge) were not available in the administrative dataset used for this study.

Interpreter support was available when needed through the Ministry of Health’s national support hotline, providing telephone-based interpretation services. Since interpreter services were delivered externally and on-demand, detailed operational information such as the number of interpreters, working hours, languages covered, and visit-level utilization could not be quantified within the scope of this study.

### 2.4. Data Analysis

Data were analyzed using IBM SPSS Statistics software (Version 27.0, IBM Corp., Armonk, NY, USA). Descriptive statistics (frequency, percentage, mean, and standard deviation) were used to summarize the demographic and clinical characteristics of the participants. There were no missing values in the variables included in the analysis. The variables of gender, nationality, admission period, diagnosis group, laboratory tests, consultations, and outcome were presented with frequency and percentage values. Seasonal and annual admission distributions were shown with histograms. Consultation clinics with fewer than 100 encounters during the study period were grouped under the ‘Other’ category, which included departments such as dermatology, neurosurgery, psychiatry, etc. This categorization was performed to maintain analytical clarity and avoid fragmentation due to very low-frequency subgroups.

## 3. Results

Table 1 shows that 66.4% of the individuals participating in the study were female and 33.6% were male. Syrians constitute half (50%) of the participants, followed by individuals of Azerbaijani nationality (15.2%), Afghan nationality (10%), Iraqi nationality (8.7%), Iranian nationality (6.1%), and German nationality (3.1%). This distribution shows that the sample consists largely of individuals of Middle Eastern origin. The average age of participants was 31.9 ± 14.4 for women and 26.8 ± 18.2 for men.

Table 2 shows that the highest percentage of applications was in spring (30.1%), while the lowest was in winter (17.9%). In terms of annual distribution, ED visits increased overall from 2020 to 2024, peaking in 2024 (22.1%). The lower frequency observed in 2025 (2.7%) reflects partial-year data, as the study period included only the first months of 2025.

**Figure 1:**
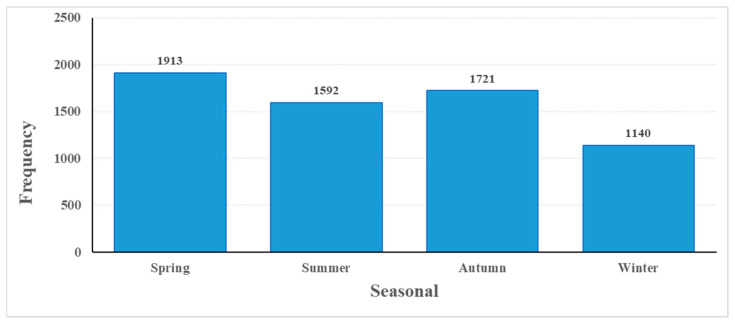
Histogram showing the seasonal distribution of emergency department visits.

Figure 1 shows the seasonal distribution of a total of n = 6366 cases visiting the emergency department between 2020 and 2025. Analysis of the graph shows that the highest number of visits occurred in spring, while the lowest number occurred in winter.

**Figure 2:**
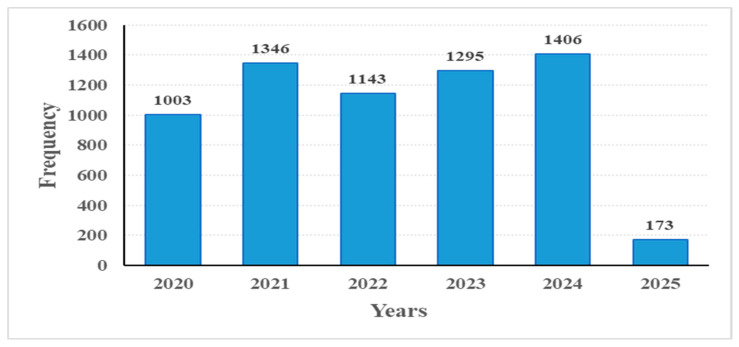
Histogram showing the distribution of emergency department visits by year.

Figure 2 shows the distribution of the total number of n = 6366 cases visiting the emergency department between 2020 and 2025 by year. The average value, based on the daily number of patients, was determined to be 3.2 ± 1.41.

Table 3 shows that 99.3% of cases were walk-in visits, while 0.7% were critical cases transported by ambulance. Looking at the diagnosis groups, the highest number of admissions occurred in the fields of internal emergencies (36.1%) and genitourinary emergencies (32.2%), followed by surgical (16.3%) and neurological (7.7%) emergencies. The laboratory test rate was relatively low; laboratory tests were not performed in 90.5% of cases. Consultations were requested in 48.1% of participants, with the most frequent referrals being to gynecology and obstetrics (21.2%), followed by other departments (12.5%) and ophthalmology (3.9%). Upon examining observation times, it was found that 76.9% of patients remained under observation for more than 8 h. This finding may reflect multiple system-level factors influencing ED length of stay. According to the referral outcomes, 92.9% of cases were discharged, 6.8% were admitted, and only 0.2% were transferred

Table 4 shows that seasonal variation was noted across diagnostic categories. Chest and cardiac emergencies were most frequent in autumn (n = 74, 28.2%) and winter (n = 69, 26.5%), and least frequent in summer (n = 48, 18.4%). Genitourinary (n = 2052) and internal emergencies (n = 2295) constituted the largest diagnostic groups, both peaking in winter (genitourinary: n = 551, 26.9%; internal: n = 627, 27.3%). Infectious disease emergencies occurred most commonly in autumn (n = 67, 29.7%), while neurological emergencies peaked in spring (n = 146, 29.5%). Surgical emergencies showed a marked increase during summer (n = 332, 32.0%). Regarding disposition, discharge predominated across all categories; admission rates were highest in surgical emergencies (10.9%, n = 113) and infectious disease emergencies (10.6%, n = 24), followed by chest and cardiac emergencies (9.2%, n = 24). Referrals were rare overall and were mainly observed in genitourinary (0.3%, n = 4), infectious disease (2.7%, n = 6), and neurological emergencies (0.6%, n = 3).

## 4. Discussion

In this retrospective study, we described the emergency department utilization profile of foreign nationals presenting to a tertiary university hospital in Balıkesir, Türkiye, between 2020 and 2025. When the demographic characteristics of the patient profile were examined, it was found that the number of female patients was higher than that of male patients. To contextualize this finding, official statistics for Balıkesir province indicate that the registered foreign population consists of 11,968 individuals, of whom 5837 (48.8%) are female and 6131 (51.2%) are male [14]. Compared with this population structure, the proportion of female ED visits in our cohort (66.4%) appears substantially higher than expected, suggesting that female foreign nationals may utilize ED disproportionately more than males. Similarly, the literature reports that the proportion of female patients may be high in refugee populations [15]. Another study conducted in Düzce province in Türkiye found that 52% of foreign national patients were female [16]. This situation may stem from the fact that women and children have priority in accessing health services, especially during war and migration processes. Therefore, while the gender distribution favors women in peaceful foreign national communities, this balance may change in special circumstances such as war. The female-dominated gender ratio in our study points to the presence of a foreign national population arriving with family reunification and the distinctiveness of women in seeking healthcare services.

In terms of seasonal distribution, foreign national patient visits have been observed to increase in the spring and summer months. This increase parallels the seasonal trends observed in emergency department visits across Türkiye [17]. In our series, the concentration of foreign national patients in the spring–summer period may be related to the agricultural labor mobility in our region and summer tourism, as well as the increase in travel during the lifting of COVID-19 restrictions. This finding indicates that seasonal fluctuations should be taken into account in healthcare planning. We also observed an overall increase in the number of foreign national ED visits between 2020 and 2024. This upward trend may reflect increasing migration-related population dynamics in the region, as well as changes in healthcare-seeking behavior and access patterns over time. Seasonal differences were also found between diagnostic categories and discharge/hospitalization outcomes. The increase in the frequency of certain diagnostic groups during specific seasons suggests, as noted in the literature, that emergency department demand may be related to seasonal factors and demographic fluctuations [18]. Furthermore, differences in hospitalization rates among diagnostic categories indicate that many visits can be managed at the outpatient level; however, selected diagnostic groups appear to contribute disproportionately to inpatient demand and consultation workload. This observation is consistent with the findings of a systematic review of differences in emergency department utilization behaviors among foreign populations, highlighting that foreign nationals’ access pathways and barriers to accessing the health system may influence emergency department utilization patterns [5]. Overall, these findings underscore the importance of seasonal and diagnosis-specific emergency department resource planning and reveal that strengthening staffing strategies, interpreter/communication support, and coordination with inpatient departments during peak periods are critical to maintaining quality of care and patient safety. When the types of visits were evaluated, it was found that the vast majority of foreign national patients visited the emergency department on an outpatient basis, while the rate of arrivals by ambulance remained low. This finding is consistent with the general trend of foreign nationals visiting emergency departments in our country [16]. Similarly, a study of refugee patients visiting the pediatric emergency department in Ankara/Türkiye reported that 78–80% of visits were walk-ins [19]. In our study, almost all foreign national patients came to the ED on their own. One possible reason why the foreign national population prefers to go directly to the hospital rather than using ambulance services to access emergency healthcare may be the lack of interpreter services in ambulance services, which are available in hospitals. This highlights the importance of translation services and referral mechanisms for foreign nationals in the healthcare provided to them.

Our study revealed noteworthy findings in terms of diagnosis and resource utilization. Low diagnostic resource utilization among foreign nationals may indicate that many ED presentations were related to low-acuity conditions that could potentially be managed in primary care settings. Beyond clinical acuity, this pattern may also reflect barriers to timely access to outpatient services, limited health literacy, and system navigation difficulties foreign nationals. Indeed, a large study conducted in Adıyaman province in Türkiye in 2015 found that 68.5% of Syrian refugee visits to the emergency department were “inappropriate,” meaning they did not require urgent medical attention [20]. Another study, a retrospective study conducted in a tertiary ED, also reported that the vast majority of patients who presented were outpatients with low urgency, indicating that EDs were being used as an alternative to primary health care services [21]. These findings support the low examination rates in our study. Therefore, the rate of visits for simple complaints that do not require advanced examination is quite high among foreign national patients. Similarly, our data show that the vast majority of foreign national patients were discharged by the ED physician with symptomatic treatment. In light of these data, it can be said that the foreign national population mainly uses the ED as an alternative to primary health care, and therefore, mild patients who require fewer diagnostic procedures are reflected in the ED. Nevertheless, because the diagnostic aggregation was designed for descriptive reporting and did not include standardized triage acuity levels, repeated visits, or formal definitions of avoidable ED use, inappropriate utilization cannot be directly measured in our dataset and should be interpreted cautiously.

Another important finding of our study is that the average time spent in the emergency department by foreign national patients is relatively long. In other words, these patients tend to be kept under observation in the emergency department for extended periods. The literature also reports similar findings indicating that refugee patients may spend longer time in EDs. In a study by Gulacti et al. (2017) in Adıyaman, the average length of stay in the emergency department for Syrian patients was significantly longer than for other patients (*p* < 0.001) [20]. Prolonged emergency department stays may be related to various reasons: factors such as slowing down of the anamnesis and treatment process due to language barriers and communication difficulties, lack of social support and discharge planning, and situations requiring some patients to be kept under observation for a certain period of time, such as trauma. Although language barriers and communication difficulties may contribute to prolonged observation, alternative explanations should also be considered, including ED crowding, limited inpatient bed availability, delays in consultation and admission processes, administrative procedures related to registration and insurance coverage, and changes in workflow during the COVID-19 pandemic (e.g., isolation and testing protocols). Indeed, communication problems are identified as a significant barrier in refugee care [22]. Although this rate remained lower in our study, thanks to telephone-based interpreter support via the Ministry of Health national hotline, it is clear that the language barrier has not been completely eliminated. However, it should also be noted that a review of the international literature shows that a similar trend is not seen in every HCS. For example, a recent study conducted in the United States indicated that refugee children were more likely to seek care (24.4% vs. 22.1%), but there was no difference in the level and duration of care provided in the ED [23]. It is thought that the comprehensive insurance and interpretation services provided to refugee families in the United States keep emergency department processing times close to those of the native population. In contrast, the long emergency department stay times of foreign national patients in our country [24]; indicate that the process of adaptation to the HCS is ongoing and that there is room for improvement in service delivery.

Our treatment and outcome data also paint a consistent picture when compared with the literature. The high discharge rate observed in this cohort is consistent with is similar to recent studies in the literature [25,26]. This rate confirms that the majority of emergency department visits involve conditions that can be treated on an outpatient basis. Our findings show that a significant proportion of ED visits among the foreign national population can be resolved there. The low admission rate suggests that foreign national patients primarily use the emergency department as an outpatient clinic. This may be due to difficulties in accessing primary care or routine specialist consultations. Indeed, refugees and foreign nationals often use EDs as a “safety net” during the initial stages of adapting to a country’s HCS after settling there [23]. Studies conducted in other regions of Türkiye have shown that one-third of all hospital examinations of foreign national patients take place in the ED, that this rate is significantly higher than that of the native population, and that the ED outpatient clinic is the unit most frequently used by foreign nationals over a five-year period (15.6% of all visits) [15,16]. These findings underline the need to adapt emergency care delivery to migration-related patient profiles. Implementing culturally competent care models, strengthening interpreter availability, and developing ED-to-primary care referral pathways may reduce avoidable ED utilization. At the policy level, improving foreign nationals’ access to primary healthcare services and clarifying administrative procedures may help shift low-acuity visits away from EDs and ensure more equitable and efficient healthcare delivery.

## 5. Conclusions

Based on data from the Emergency Department of Balikesir University Hospital tertiary (third-level) referral center, this study revealed that the profile of foreign national patients presenting to this setting over the past five years has several distinctive characteristics. Foreign national patients’ ED visits exhibit characteristic features, including seasonal variability, a female-dominated gender distribution, a predominance of outpatient visits, low use of diagnostic tests, prolonged observation time, and a low admission rate. Our findings are generally consistent with the national literature and confirm the ongoing trends in foreign nationals’ health in Türkiye. In conclusion, knowledge of the demographic and clinical characteristics of foreign national patients is critical for planning ED services according to the needs of this population. Although these aspects were not directly measured in our dataset, strengthening communication support (e.g., interpreter services) and culturally competent care may improve the quality and effectiveness of ED services for foreign nationals, as suggested in the literature. Furthermore, facilitating foreign nationals’ access to primary health care and implementing programs to improve health literacy can reduce inappropriate ED visits, allowing more efficient allocation of resources to patients with genuine emergencies. As the results show, since EDs are critical points of first contact for the health problems of foreign national populations, the resource planning and staff training of these units should be updated, taking migration dynamics into account. Our data sheds light on efforts to adapt to the increasingly diverse patient profile in our country and provides a basis for larger-scale studies in the future. As regulations regarding migrant health are implemented in health policies, the role of emergency departments is central, as the findings of this study show, and it would be appropriate to take corrective steps within this framework. Thus, both the health needs of the foreign nationals can be met, and the overall efficiency of the health system can be increased.

### 5.1. Strengths of the Study

The most important strength of this study is its large dataset, which includes 6366 foreign national patients who visited the Emergency Department of Balikesir University Hospital between 2020 and 2025. This comprehensive six-year data set has enabled the presentation of a detailed profile of foreign nationals, covering their demographic characteristics, diagnoses, and treatment processes. The census (complete enumeration) approach ensured that all eligible ED visits within the study period were included, thereby fully representing the study population and filling an essential gap in region-specific evidence on emergency care utilization among foreign nationals. The research provided valuable data for emergency medicine practice and public health planning by offering comprehensive insights into the use of the emergency department by foreign national patients.

### 5.2. Limitations of the Study

This study has several limitations. First, this was a single-center study conducted in a tertiary referral hospital; the findings primarily reflect the case mix and care processes of a third-level emergency department and may not be generalizable to other settings. Second, the study population included only foreign nationals, and no comparison group of Turkish citizens was available; thus, direct comparisons between foreign nationals and native patients regarding ED utilization patterns, diagnoses, or outcomes could not be performed. Third, heterogeneous subgroups within the foreign patient population (e.g., refugees, asylum seekers, tourists, and seasonal workers) could not be differentiated using the available dataset, limiting subgroup-specific interpretations. Fourth, the retrospective design may be subject to missing or inaccurate records and did not allow assessment of post-discharge outcomes or long-term follow-up. Mortality outcomes could not be evaluated because in-hospital and post-discharge mortality data were not available in the dataset. In addition, although telephone-based interpreter support was available through the Ministry of Health’s national support hotline, interpreter utilization (e.g., frequency of use, languages requested, response times) was not recorded in the administrative dataset and therefore could not be evaluated. Finally, the COVID-19 pandemic during the study period may have influenced ED utilization patterns, complicating the interpretation of temporal and seasonal trends.

### 5.3. Recommendations

The results emphasize the importance of enhancing emergency healthcare services for foreign national patients by improving communication support, boosting cultural competence among healthcare staff, and clarifying administrative procedures for individuals with different residency or citizenship statuses. Hospitals should consider developing protocols that specifically address the needs of various foreign national groups, especially during times of increased healthcare demand, such as pandemics. Furthermore, local health authorities could use the demographic and clinical patterns identified in this study to better allocate resources and staff, and to plan services, in EDs serving foreign national populations.

Future studies should use multicenter and comparative designs across various regions to evaluate how broadly these findings apply and to enable meaningful comparisons between foreign national patients and Turkish citizens. Research focusing on subgroup analyses such as refugees, tourists, asylum seekers, and seasonal workers is also needed to better understand the different healthcare utilization patterns within the diverse foreign national population. Prospective or longitudinal studies examining post-discharge outcomes would further enhance understanding of the long-term health trajectories of foreign national patients. Such studies could help identify risk factors, unmet needs, and gaps in continuity of care that are not apparent in retrospective ED-based analyses.

## Figures and Tables

**Table 1:** Descriptive information about the individuals participating in the study.

Variables		n	%
**Gender**	Female	4228	66.4
	Male	2138	33.6
**Nationality**	Syria	3183	50.0
	Afghanistan	636	10.0
	Germany	199	3.1
	Azerbaijan	970	15.2
	Iraq	556	8.7
	Iran	389	6.1
	Other	433	6.8
**Variables**		**Mean**	**S.D.**
**Age**	Female	31.9	14.4
	Male	26.8	18.2

S.D.: Standard Deviation.

**Table 2:** Distribution of hospital visits by season and year.

Variables		n	%
**Season**	Spring	1913	30.1
	Summer	1592	25.0
	Autumn	1721	27.0
	Winter	1140	17.9
**Year**	2020	1003	15.8
	2021	1346	21.1
	2022	1143	18.0
	2023	1295	20.3
	2024	1406	22.1
	2025	173	2.7

**Table 3:** Participant arrival method and clinical information.

Variables		n	%
**Arrival Method**	Ambulance	43	0.7
	Outpatient	6323	99.3
**Diagnosis**	Internal Emergencies	2295	36.1
	Genitourinary Emergencies	2052	32.2
	Infectious Disease Emergencies	226	3.6
	Chest and Cardiac Emergencies	261	4.1
	Neurological	493	7.7
	Surgical Emergencies	1039	16.3
**Laboratory Tests**	No	5759	90.5
	Yes	607	9.5
**Consultation**	No	3305	51.9
	Yes	3061	48.1
**Consultation Clinic**	No Consultation	3311	52.0
	Orthopedics and Traumatology	146	2.3
	Otolaryngology	200	3.1
	Obstetrics and Gynecology	1347	21.2
	Internal Medicine	195	3.1
	Ophthalmology	248	3.9
	Pediatrics	125	2.0
	Other	794	12.5
**Observation Period (hours)**	8 h and Under	1469	23.1
	Over 8 h	4897	76.9
**Result**	Admission	436	6.8
	Discharged	5917	92.9
	Referral	13	0.2

**Table 4:** Distribution of diagnostic categories by season and disposition.

Variables	Seasons								Dispositions					
Diagnosis Category	Spring		Summer		Autumn		Winter		Admitted		Discharged		Referral	
	n	%	n	%	n	%	n	%	n	%	n	%	n	%
**Chest and Cardiac** **Emergencies**	70	26.9	48	18.4	74	28.2	69	26.5	24	9.2	237	90.8	0	0
**Genitourinary** **Emergencies**	481	23.3	498	24.3	522	25.5	551	26.9	144	7	1904	92.7	4	0.3
**Infectious Disease** **Emergencies**	60	26.6	46	20.3	67	29.7	53	23.4	24	10.6	196	86.7	6	2.7
**Internal** **Emergencies**	454	19.8	615	26.8	599	26.1	627	27.3	108	4.7	2187	95.3	0	0
**Neurological** **Emergencies**	146	29.5	117	23.8	113	22.9	117	23.8	28	5.7	462	93.7	3	0.6
**Surgical** **Emergencies**	252	24.2	332	32.0	195	18.8	260	25.0	113	10.9	926	89.1	0	0

## Data Availability

The raw data supporting the conclusions of this article will be made available by the authors on request.

## Abbreviations

The following abbreviations are used in this manuscript:

ED	Emergency Department
HCS	Healthcare System
